# Supplementary material for: PCM1 coordinates centrosome asymmetry with polarized endosome dynamics to regulate daughter cell fate
Source: Nat Commun. 2025 Nov 28;16:10728. doi: 10.1038/s41467-025-65756-2 (PMC12663461; doi:10.1038/s41467-025-65756-2)
Supplement: Supplementary file 1 — Supplementary Information [file 41467_2025_65756_MOESM1_ESM.pdf]

Supplementary Information (Zhao et al.)

Supplementary Figures and Figure Legends

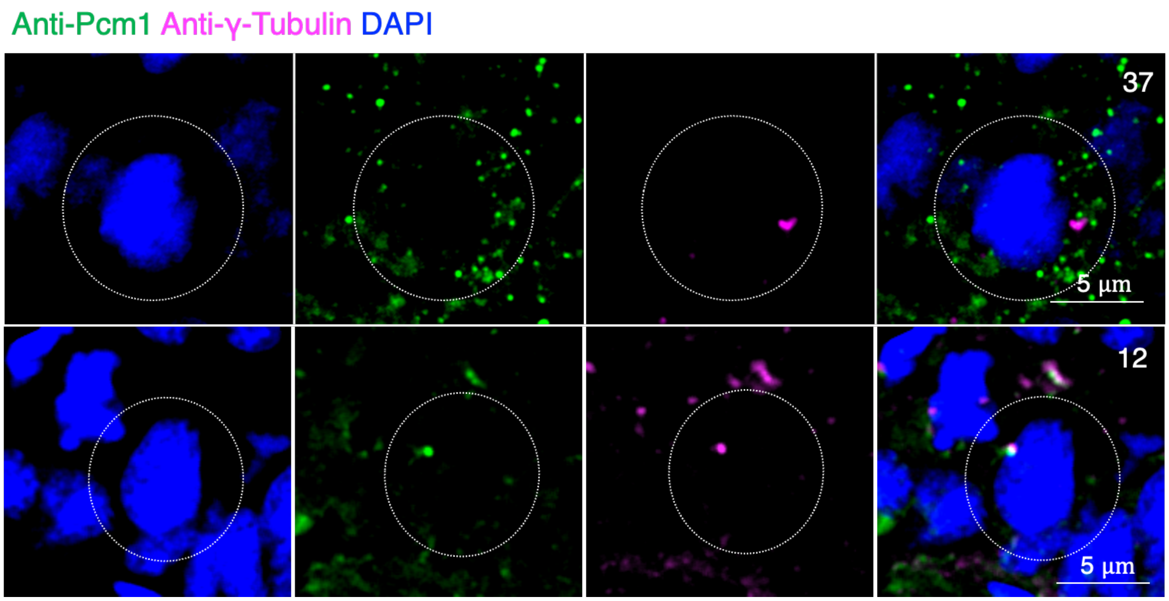

Supplementary Fig 1. Pcm1 distribution patterns in zebrafish RGPs at interphase. Interphase RGPs with pericentriolar enrichment (upper row, n=37), and tight centrosomal association (lower row, n=12). RGPs were from 6 embryos of 28-32 hpf. Scale bars, 5 μm.

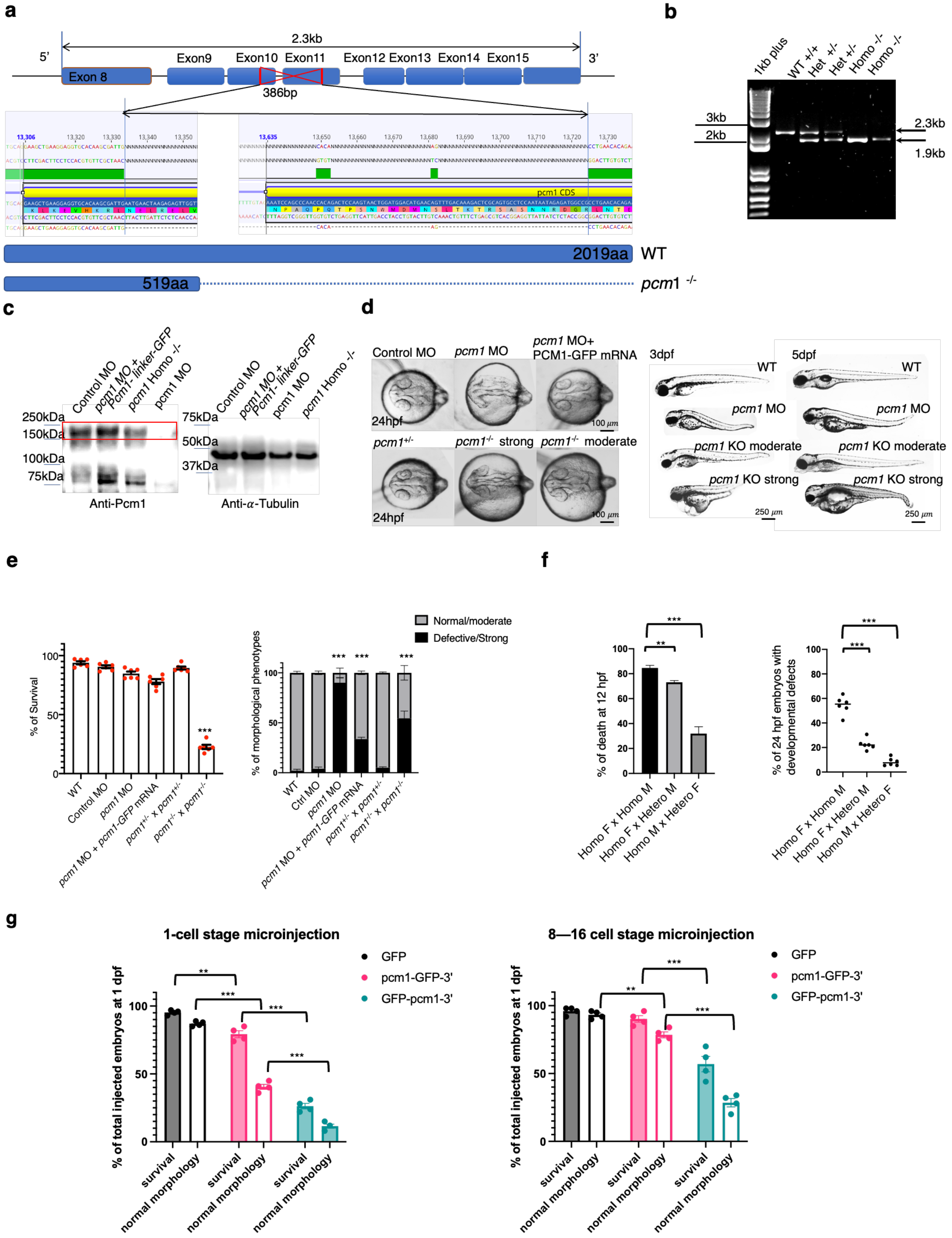

Supplementary Fig 2. Generation of CRISPR-Cas9 *pcm1* KO and characterization of its developmental defects.

a. A schematic showing the genomic lesion in the *pcm1* locus. The DNA sequence and translated amino acid sequence (Genious Prime 2022) show a 386 bp deletion between Exon 10 and 11 in the *pcm1* KO. The predicted truncated Pcm1 protein is 519 a.a., which is much smaller than WT Pcm1 (2019 a.a.). **b**. Gel electrophoresis of RT-PCR products shows shortened bands from the *pcm1* mutant. The primers flank Exon 8 and Exon 15 as shown in (a). **c**. Western blot with anti-Pcm1 and anti-α-Tubulin antibodies using 1 dpf embryonic extracts. **d**. Dorsal views of 24 hpf embryonic brains (left) and lateral views of 3 and 5 dpf larvae showing the developmental defects of *pcm1* KO mutant compared to control and *pcm1* MO groups. **e**. Statistics of survival and morphological deformity. pcm1 MO and pcm1 KO mutants from homozygous mutant parents showed significantly decreased survival and increased morphological defects at 24 hpf compared to control groups and embryos derived from heterozygous parents. Six independent experiments were performed. Unpaired two-tailed t test, n =6, *** $p < 0.001$. Error bars indicate SD. **f**. Compensation by the parental *pcm1* gene products. Both maternal and paternal *pcm1* gene products contribute to decreased death and developmental defects. Six independent experiments were performed. Unpaired two-tailed t test, n =6, *** $p < 0.001$; ** $p < 0.01$. Error bars indicate SD. **g**. Statistic of embryonic survival with normal morphology in groups injected with GFP mRNA, pcm1-GFP mRNA, and GFP-pcm1 mRNA at 1-cell stage or 8-16 cell stage. Both one-cell stage and 8-16-cell stage microinjections showed that GFP-pcm1 mRNA injected larvae had significantly lower survival and normal morphology than GFP mRNA injected controls and pcm1-GFP mRNA injected groups. Most pcm1-GFP mRNA-injected embryos survived, and almost all survived embryos were normal when the microinjections were done at the 8-16 cell stage. Unpaired t test, four independent repeats with 50 embryos per group of each injection. *** $p<0.001$, t-test, two-tailed unpaired. Error bars indicate SD.

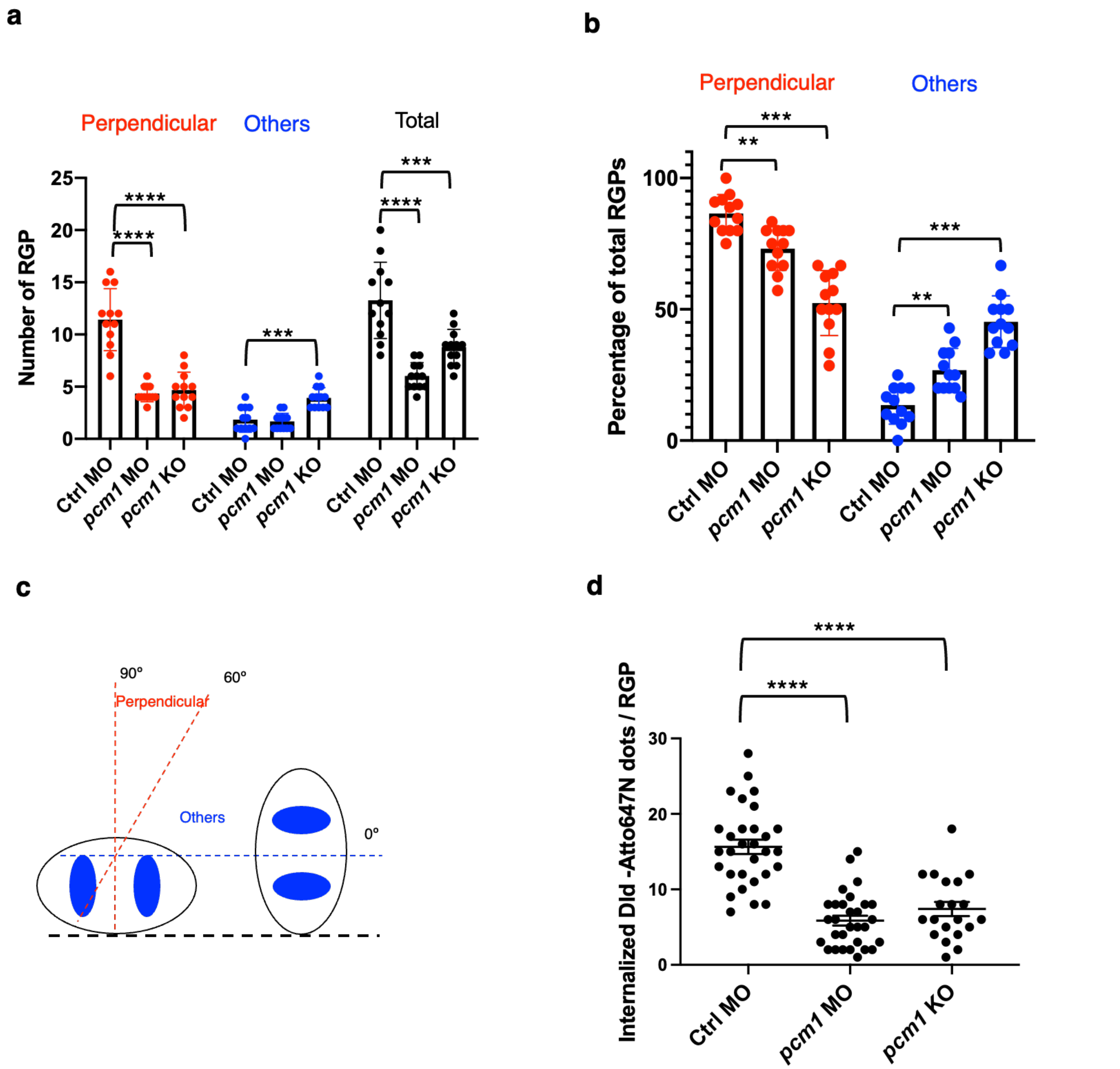

Supplementary Fig 3. PCM1 knockdown and knockout decrease mitotic RGPs and internalized Dld signals in the developing forebrain.

a. Quantification of mitotic RGPs in the developing forebrain of 24 hpf embryos during 30 min time-lapse imaging. 12 embryos from four independent experiments were included in each group. Both *pcm1* MO and *pcm1* KO embryos showed significantly fewer mitotic RGPs than the control. They also showed decreased perpendicularly dividing RGPs and a corresponding increase of non-perpendicularly dividing RGPs compared to control. Unpaired two-tailed t test, **** $p < 0.0001$; *** $p < 0.001$; n = 12 embryos per group. Error bars indicate SD. **b**. Statistics of the ratio of perpendicularly dividing RGPs and others in control MO, pcm1 MO, and pcm1 KO sample groups. Unpaired two-tailed t test, *** $p <$

0.001; ** $p < 0.01$; n = 12 embryos per group. Error bars indicate SD. **c.** Schematic map of perpendicularly dividing RGPs (dividing plane is 60°-90° to the ventricular surface) and non-perpendicularly dividing RGPs (others, dividing plane is < 60° to the ventricular surface). The black dashed line denoted the ventricular surface (0°). **d**. Quantification of internalized Dld-Atto647N particles in mitotic RGPs. Mitotic RGPs from both *pcm1* MO and *pcm1* KO embryos showed a significantly decreased number of Dld-Atto647N particles compared to the control. For the control MO and *pcm1* MO group, n = 30 RGPs from 6 embryos. For *pcm1* KO, n = 20 RGPs from 6 embryos. Unpaired two-tailed t test, **** $p < 0.0001$. Dld-Atto647N particles in the RGP at anaphase were counted for quantification. Error bars indicate SEM.

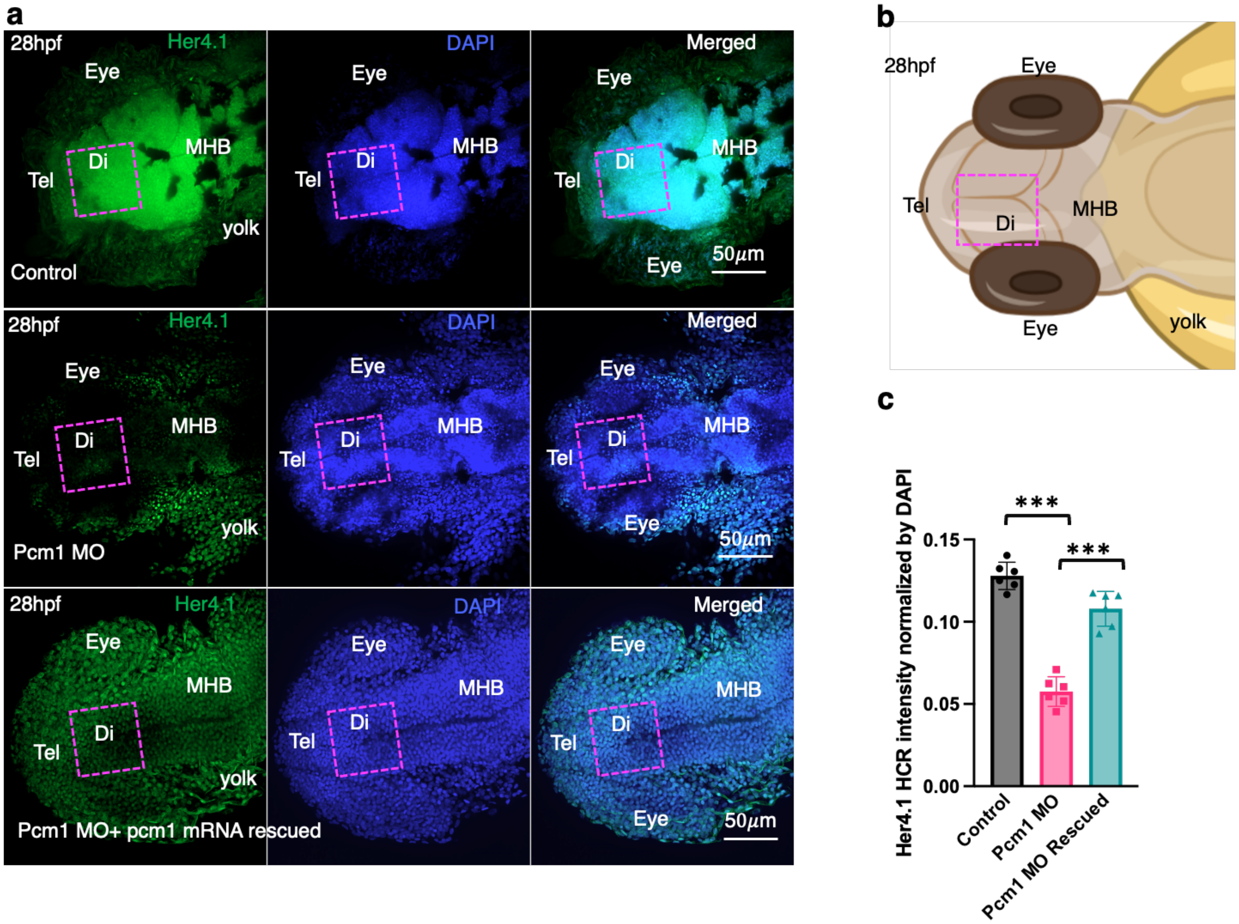

Supplementary Fig 4. **Reduced *her4.1* expression in *pcm1*-deficient embryos**. **a**. Images show *her4.1* HCR in situ in 28 hpf embryos. In the panel, images of max projection of 20 z-plane in the dorsal forebrain were shown. Her4.1 probes were detected by HCR™ Gold Amplifier X3 488 (green). Nuclear counterstaining was applied with DAPI (blue). The magenta rectangle areas marked in each sample indicate the forebrain ventricle zone in the 28 hpf embryonic brain shown in (b), which was used for

quantification in (c). **b**. The cartoon figure shows the main anatomical structures of the 28 hpf embryonic brain shown in (a). Created in BioRender. Zhao, X. (2025) https://BioRender.com/ps32kwp . Di, Diencephalon; MHB, Midbrain-Hindbrain Boundary; Tel, Telencephalon. **c**. Statistics of *her4.1* fluorescent intensity in the HCR samples normalized by DAPI. pcm1 MO embryos show significantly less Her4.1 HCR fluorescent intensity than the control group and the rescued group. Max projection images from six embryos of each group were used for the statistics. Unpaired two-tailed t-test, *** $p<0.001$, n=6. Error bars indicate SD.

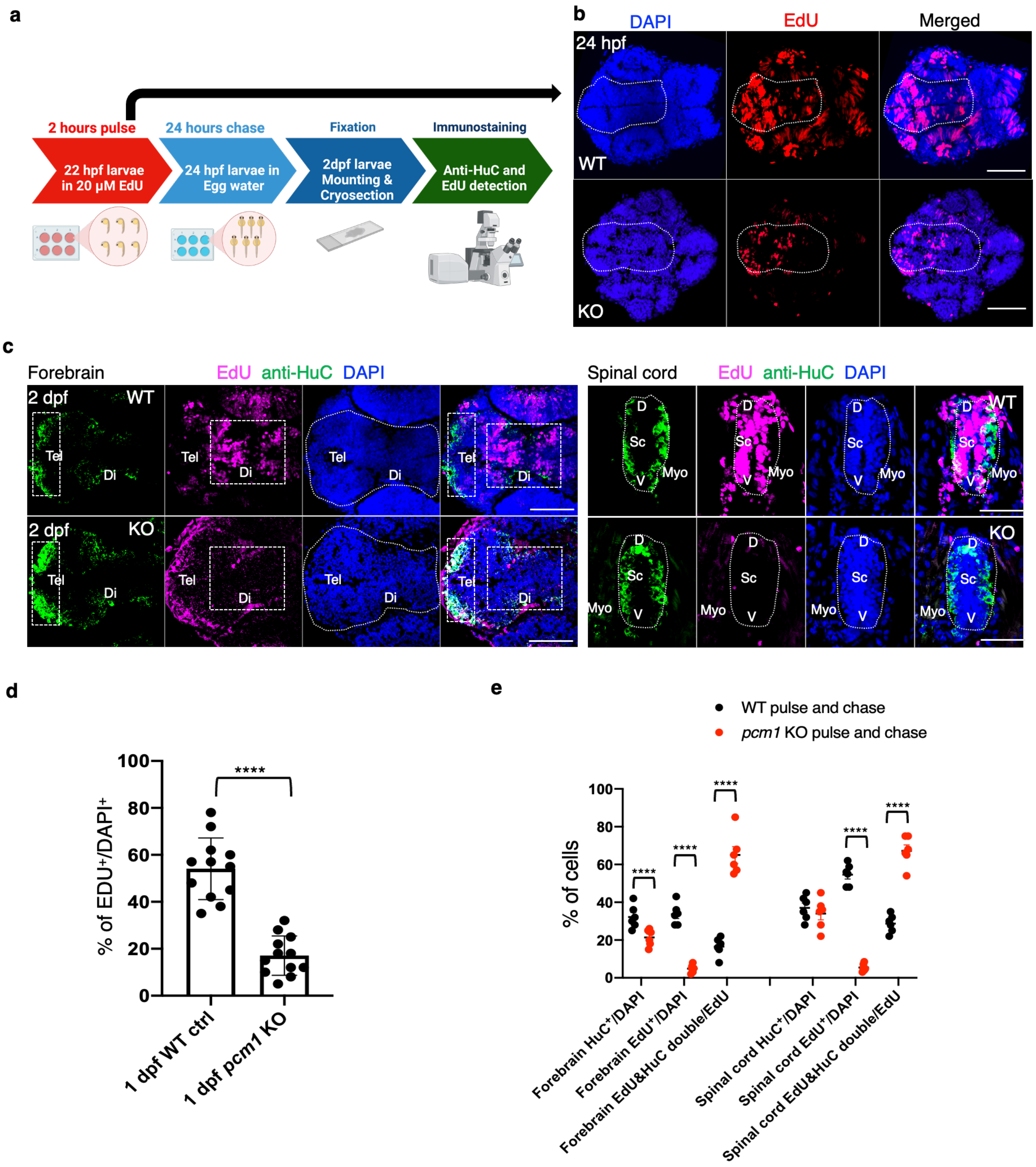

Supplementary Fig 5. Pcm1 is required for the proliferation and maintenance of progenitors revealed by EdU pulse and pulse-chase labeling. a. Schematic of EdU pulse only and EdU pulse-chase experiments. Created in BioRender. Zhao, X. (2025) https://BioRender.com/ps32kwp . **b**. Immunofluorescent images of cryo-sectioned 24 hpf embryonic forebrain following EdU pulse labeling. Scale bar, 100 μm. **c**. Immunofluorescent images of cryo-sectioned 2 dpf embryos (forebrain, left; spinal cord, right) following EdU pulse-chase labeling. D, Dorsal; Di, Diencephalon; Myo, Myotome;

Sc, Spinal cord; Tel, Telencephalon; V, Ventral. Scale bar, 50 μm. **d**. Quantification of EdU pulse labeling, shows a decreased ratio of $EdU^+/DAPI^+$ cells in 1dpf *pcm1* KO compared to control. Cryosections from 12 embryos were used for each group. **** $p < 0.0001$, two-tailed unpaired t test, n=12. Error bars indicate SD. **e**. Statistics of EdU pulse-chase labeling from 2 dpf cryosections of forebrain and spinal cord. Cryosections from 6 embryos were used for each group. **** $p < 0.0001$, unpaired two-tail t test, n=6. Error bars indicate SEM.

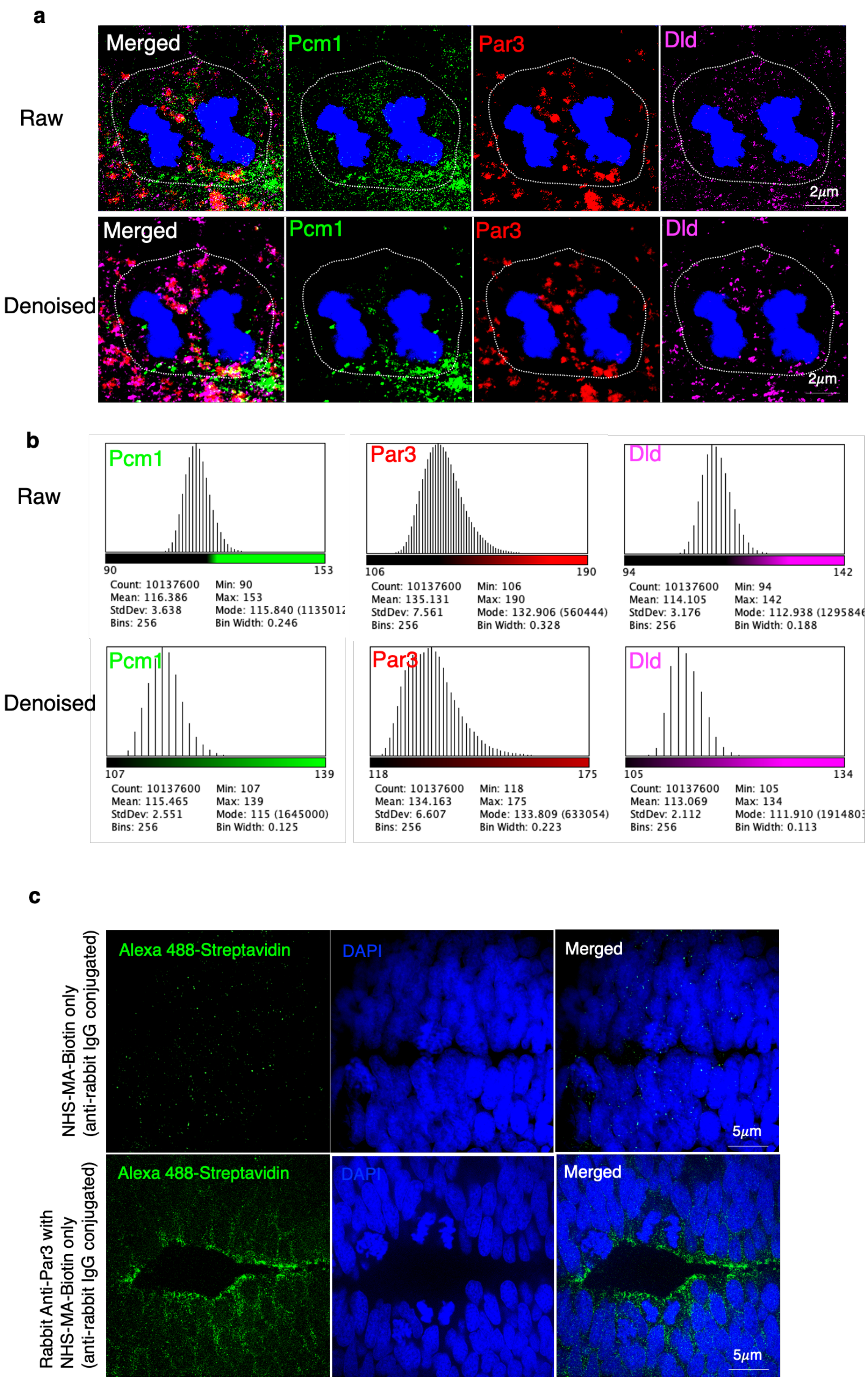
a
Raw
Denoised
Merged
Pcm1
Par3
Dld
2μm
b
Raw
Denoised
Pcm1
90
153
Count: 10137600
Mean: 116.386
StdDev: 3.638
Bins: 256
Min: 90
Max: 153
Mode: 115.840 (113501
Bin Width: 0.246
Par3
106
190
Count: 10137600
Mean: 135.131
StdDev: 7.561
Bins: 256
Min: 106
Max: 190
Mode: 132.906 (560444)
Bin Width: 0.328
Dld
94
142
Count: 10137600
Mean: 114.105
StdDev: 3.176
Bins: 256
Min: 94
Max: 142
Mode: 112.938 (1295846
Bin Width: 0.188
Pcm1
107
139
Count: 10137600
Mean: 115.465
StdDev: 2.551
Bins: 256
Min: 107
Max: 139
Mode: 115 (1645000)
Bin Width: 0.125
Par3
118
175
Count: 10137600
Mean: 134.163
StdDev: 6.607
Bins: 256
Min: 118
Max: 175
Mode: 133.809 (633054)
Bin Width: 0.223
Dld
105
134
Count: 10137600
Mean: 113.069
StdDev: 2.112
Bins: 256
Min: 105
Max: 134
Mode: 111.910 (191480
Bin Width: 0.113
c
NHS-MA-Biotin only (anti-rabbit IgG conjugated)
Rabbit Anti-Par3 with NHS-MA-Biotin only (anti-rabbit IgG conjugated)
Alexa 488-Streptavidin
DAPI
Merged
5μm

Supplementary Fig 6. Quality control for LR-ExM analysis. **a**. LR-ExM images of a mitotic RGP shown in Fig. 4 before and after Aydin denoising. Each image is the maximum intensity projection (MIP) of 20 z-planes. **b**. Histograms of each fluorescent channel of the images shown in (a) before (Raw) and after denoising (Denoised). For all three channels, the denoising process has only removed the most non-specific pixels without changing the center of peak and mean value. **c**. Endogenous biotin in LR-ExM samples. LR-ExM zebrafish brain sections stained with NHS-MA-Biotin trifunctional linker only (without any primary antibody) did not show positive signals (upper row), compared to anti-Par3 primary antibody-stained sections (lower row). Each image is the MIP of 20 z-planes.

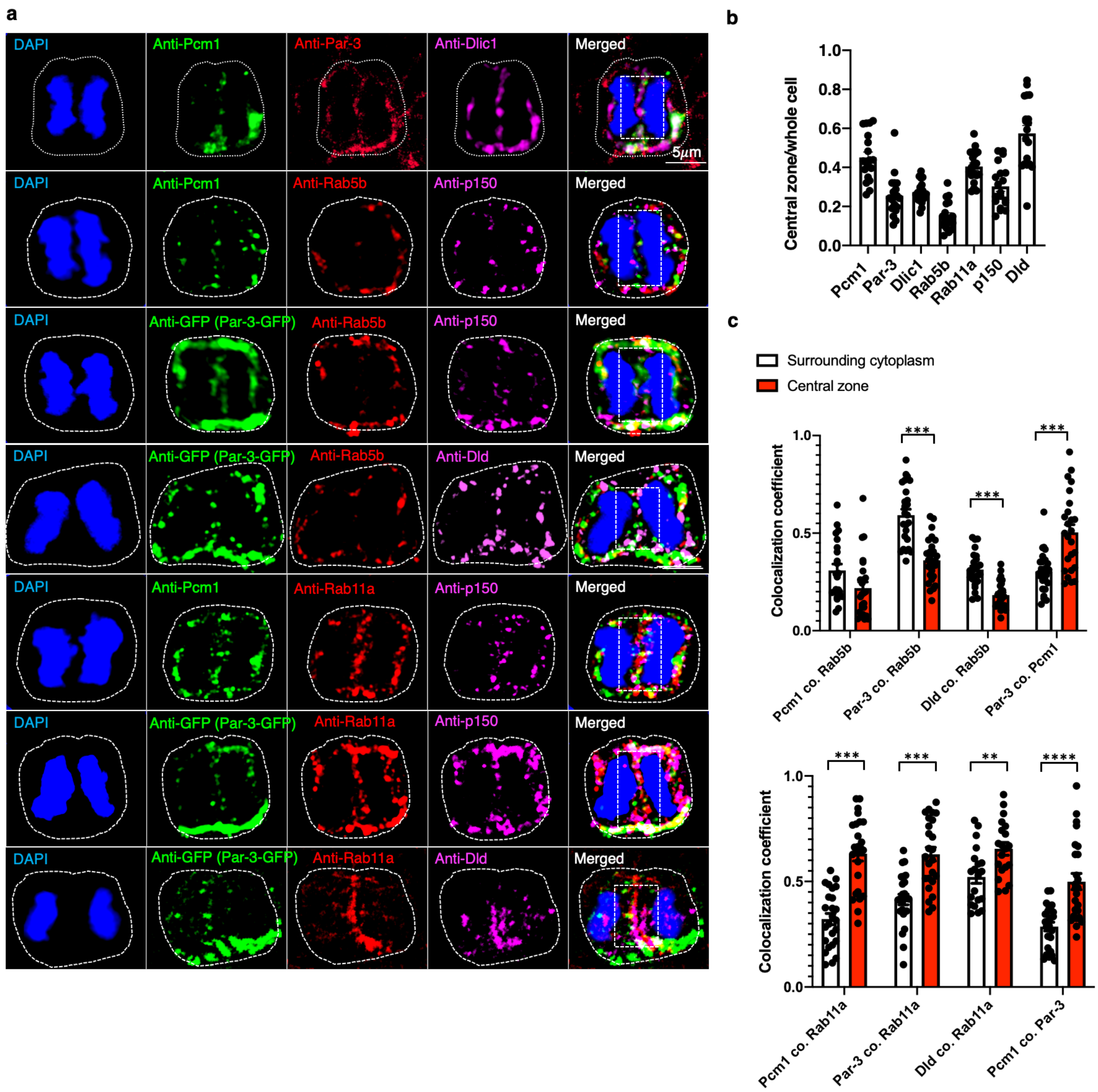

Supplementary Fig 7. Pcm1 colocalization with Par-3, endosomal, and dynein/dynactin components in anaphase RGPs via conventional fluorescent microscopy and Jacop analyses. **a**. Immunofluorescent images of Pcm1, Par-3 (or Par-3-GFP), Dld, Rab5b, Rab11a, Dlic1 and p150 in anaphase RGPs from 24 hpf embryonic forebrain. Each image is the maximum intensity projection (MIP) of 10 z-planes. The z-step is 0.26 μm. Scale bar, 5 μm. The area of the whole cell and the surrounding cytoplasm area is outlined by white dashed lines. And the outlined central rectangle (30 x 60 pixels, 1 pixel = 0.126 μm) indicates the central zone in each cell used for the analyses afterward. **b**. Statistics of protein expression in the central zone of anaphase RGPs. Pcm1, Rab11a, and Dld showed over 40% of total cell expression in the central zone, and Par-3, Dlic1, and p150 showed 20% ~ 30% of total cell expression in the central

zone. Rab5b showed less than 20% of total cell expression in the central zone. 20 cells were included in each group. Error bars indicate SD. **c**. Statistics of colocalization coefficient in the different zones of anaphase RGPs. From both charts, Pcm1 showed significantly higher colocalization with Rab11a, but not with Rab5b, in the central zone than in the surrounding cytoplasm. Par-3 and Dld showed significantly higher colocalization coefficients with Rab5b in the surrounding cytoplasm and higher colocalization coefficients with Rab11a in the central zone. Pcm1 and Par-3 showed significantly higher colocalization in the central zone than in the surrounding cytoplasm. Unpaired two-tailed t test, n=25 for each group. **** $p < 0.0001$, *** $p < 0.001$, ** $p < 0.01$; for “%PCM1 co. Rab5”, $p = 0.052445$. Error bars indicate SD.

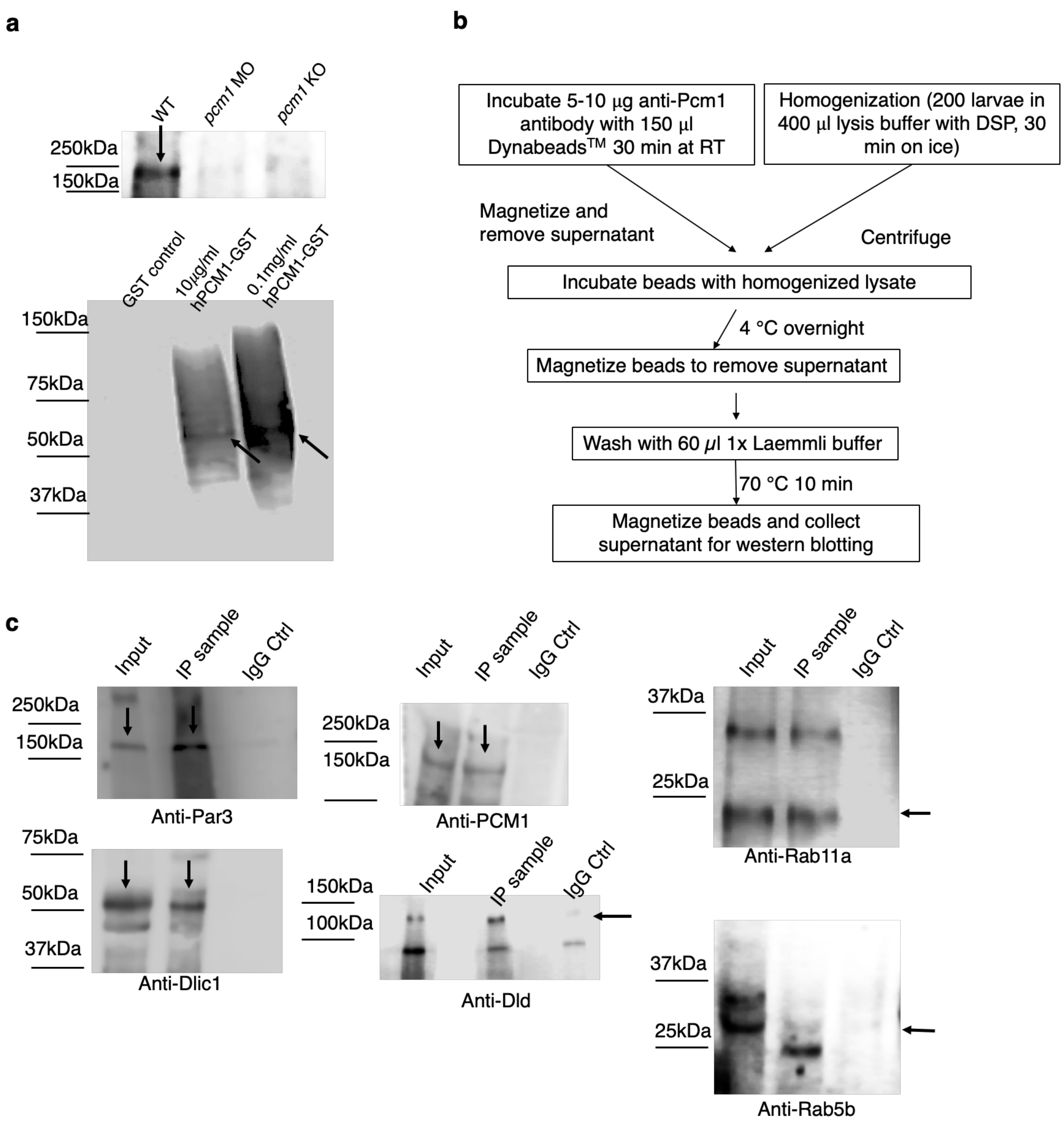

Supplementary Fig 8. *In vivo* coimmunoprecipitation of Pcm1 with Par-3, endosomal proteins, and dynein components.

a. Western blotting of 1 dpf embryonic lysate (up) and human PCM1 antigen (bottom) with the custom-generated chicken anti-PCM1 antibody. **b**. Schematic of *in vivo* Co-IP procedure. **c**. Western blotting of anti-PCM1 co-IPed samples with anti-Par-3, anti-Dld, anti-Dlic1, anti-Rab5b, anti-Rab11a and anti-PCM1 antibodies. Arrows indicate the specific band on each blot.

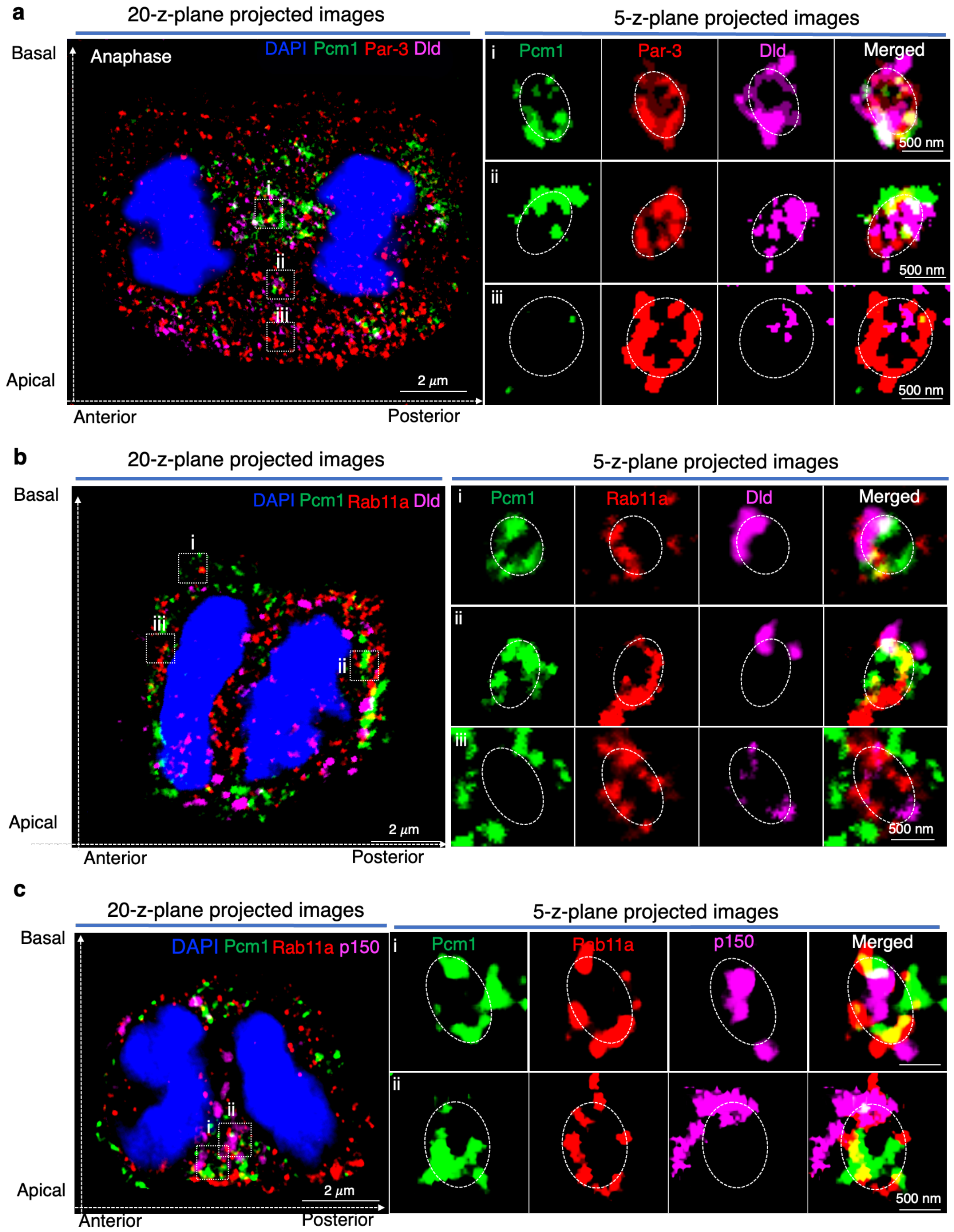

Supplementary Fig 9. Additional example LR-ExM images of Pcm1 colocalization with Par-3, Rab11a, and P150 on Dld endosomes in developing zebrafish forebrain mitotic RGPs. a. LR-ExM images of mitotic RGPs immuno-stained with anti-PCM1, anti-

Par-3, anti-Dld, and DAPI. **b**. LR-ExM images of mitotic RGPs immuno-stained with anti-PCM1, anti-Rab11a, anti-Dld, and DAPI. **c**. LR-ExM images of mitotic RGPs immuno-stained with anti-PCM1, anti-Rab11a, P150, and DAPI. 20 z plane projected whole cell images were shown on the left, and 5 z plane projected individual Dld endosomes were shown on the right. The z-step is 0.26 µm. Scale bars denote the biological size.

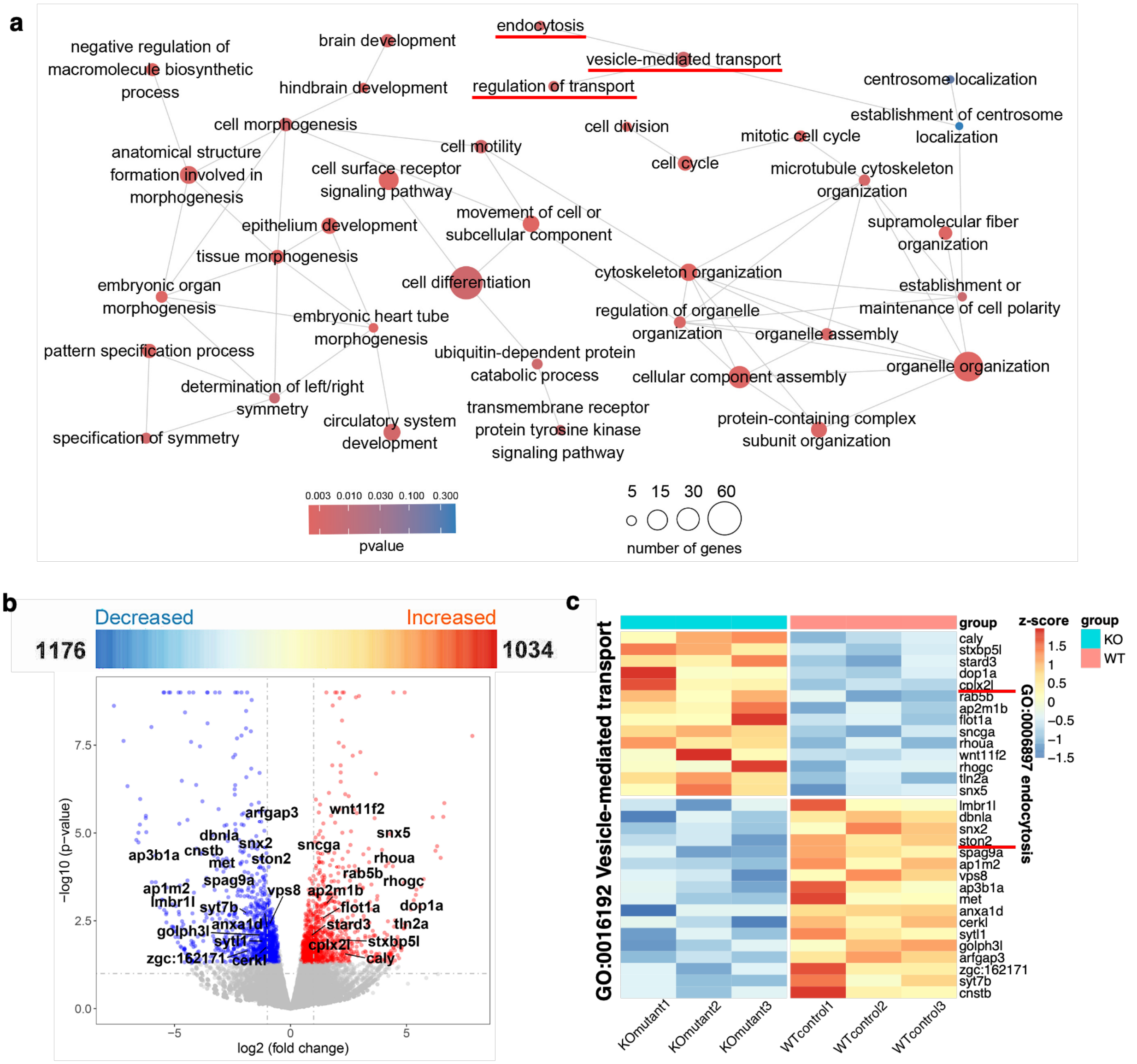

Supplementary Fig 10. Transcriptomic profiling of *pcm1* KO embryos uncovers dysregulation of genes involved in endocytosis and brain development.

a. A diagram showing a network of 37 significantly enriched GO terms of differentially expressed genes (DEGs) in *pcm1* KO embryos compared to control, built using the Cytoscape Enrichment Map ($p < 0.05$, minimal gene set size = 8). Each node represents a GO term with the color indicating P values. The size of each dot indicates the number of DEGs (5-60) for that specific GO terms. The connecting edges indicate their membership similarities. Two centrosome-related GO terms (colored in blue) were shown but they did not reach the statistical threshold of 0.05, suggesting that the role of Pcm1 in centrosomal regulation is largely non-transcriptional. **b**. Volcano plot of all genes significantly increased or decreased in *pcm1* KO compared to WT ($p < 0.05$, n = 3). The genes involved in vesicle transport, neurogenesis, and cell mitosis are shown on the plot

map. **c**. Heatmap showing the relative expression of 31 DEGs involved in endocytosis in *pcm1* KO and WT groups (z-scored).

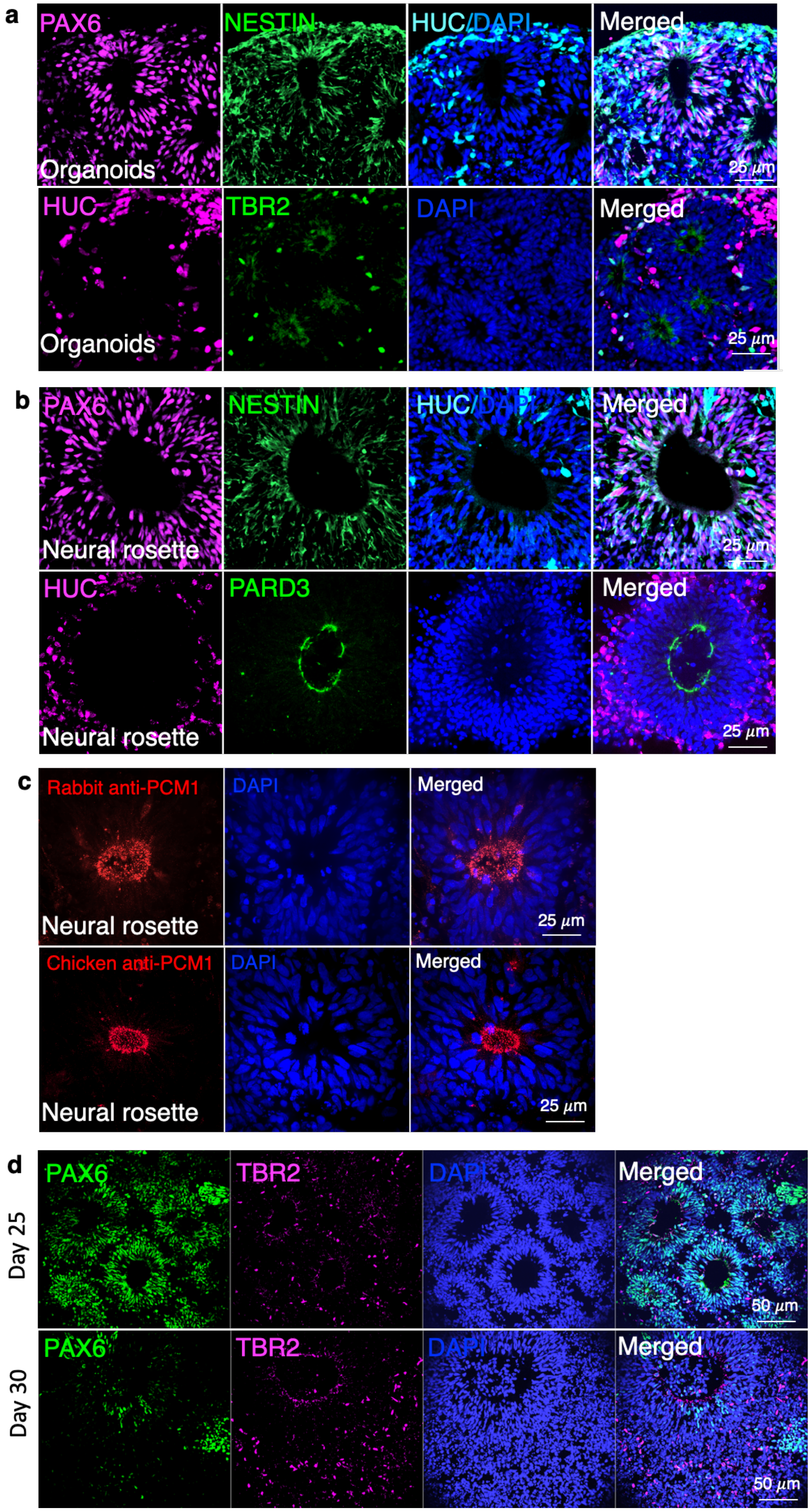
a
PAX6
NESTIN
HUC/DAPI
Merged
Organoids
25 μm
HUC
TBR2
DAPI
Merged
Organoids
25 μm
b
PAX6
NESTIN
HUC/DAPI
Merged
Neural rosette
25 μm
HUC
PARD3
Merged
Neural rosette
25 μm
c
Rabbit anti-PCM1
DAPI
Merged
Neural rosette
25 μm
Chicken anti-PCM1
DAPI
Merged
Neural rosette
25 μm
d
Day 25
PAX6
TBR2
DAPI
Merged
50 μm
Day 30
PAX6
TBR2
DAPI
Merged
50 μm

Supplementary Fig 11. Characterization of marker gene expression in hiPSC-derived forebrain organoids and neural rosettes. a. Immunofluorescent staining of cryo-sectioned forebrain organoids derived from the KOLF2.1J hiPSC line (Day 25 in culture), with anti-HuC (neuronal marker), anti-TBR2 (intermediate progenitor marker), anti-PAX6 (neural progenitor marker), and anti-Nestin (neural progenitor marker). **b**. Immunofluorescent staining of neural rosettes derived from the KOLF2.1J hiPSC line with anti-Pax6, anti-HuC, and anti-Nestin. **c.** Immunofluorescent staining showing our custom chicken anti-PCM1 antibody specificity in comparison with a previously reported rabbit anti-PCM1 antibody. Similar enriched fluorescent staining along the apical layer of neural rosettes was observed. **d**. Anti-TBR2 and anti-PAX6 double immuno-stained KOLF2.1J hiPSC forebrain organoid sections. At Day 25, most cells at the ventricle zone are anti-PAX6 positive as shown in (a). At Day 30, there were more anti-TBR2 positive cells detected in the developing SVZ (subventricular zone) and anti-PAX6 positive cells are less at the ventricular zone. Scale bar, 50 μm.

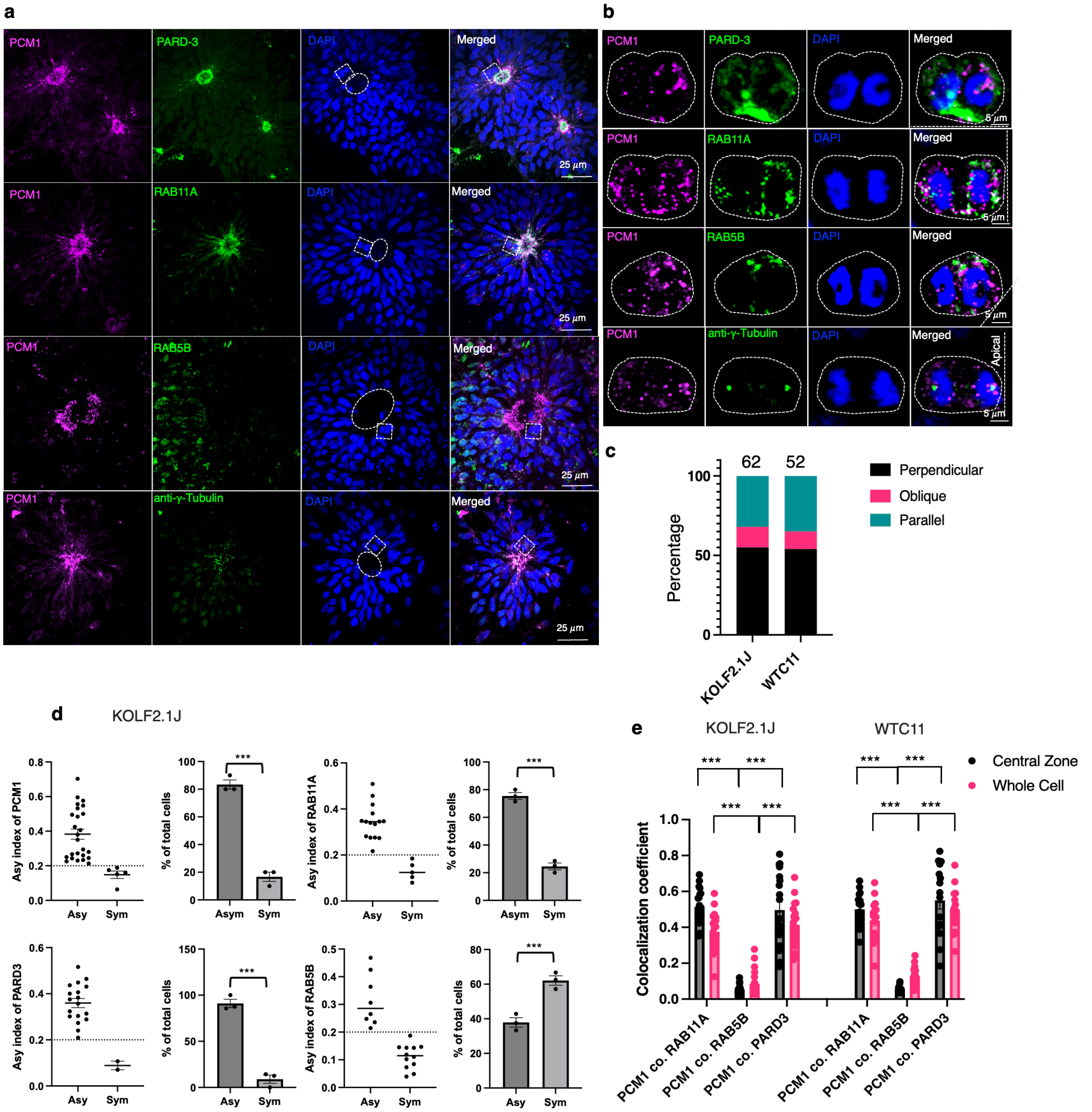

Supplementary Fig 12. PCM1 expression in mitotic neural progenitors of forebrain neural rosettes derived from hiPSCs.

a. Immunostaining of hiPSC-derived forebrain neural rosettes with anti-PCM1, PARD3, RAB11A RAB5B, and γ-Tubulin. The VZ apical layers were marked with white dashed circles. MIP of 20 z-planes was shown. Z-step is 0.26 μm. Scale bar, 25 μm. **b.** Enlarged views of mitotic NPCs marked by rectangles in (a). MIP of 10 z-planes was shown. Z-step is 0.26 μm. Scale bar, 5 μm. **c.** Statistics of division orientation. **d.** Statistics of PCM1, RAB11A, PARD3 and RAB5B asymmetric index in anaphase/telophase NPCs of

KOLF2.1J hiPSC lines derived neural rosettes. PCM1, RAB11A, and PARD3 were asymmetrically distributed in most NPCs. In contrast, RAB5B was more symmetrically distributed in most NPCs. The asymmetric index of each cell is shown. All cells are collected from the neural rosettes from three independent experiments. *** $p < 0.001$, two-tailed unpaired t-test. Error bars indicate SD. **e**. Statistics of colocalization coefficients of PCM1 with PARD3, RAB11A, and RAB5B in anaphase NPCs of forebrain neural rosettes. In the central zone, colocalization coefficients of PCM1 with RAB11A (PCM1 co. RAB11A) and PCM1 with PARD3 (PCM1 co. PARD3) are significantly higher than PCM1 with RAB5B (PCM1 co. RAB5B). The colocalization coefficients of PCM1 with PARD3 and RAB11A in the whole cell are less than the colocalization coefficients in the central zone but still significantly higher than the colocalization coefficients of PCM1 with RAB5b. *** $p < 0.001$, two-tailed unpaired t-test. n=20. Error bars indicate SD.

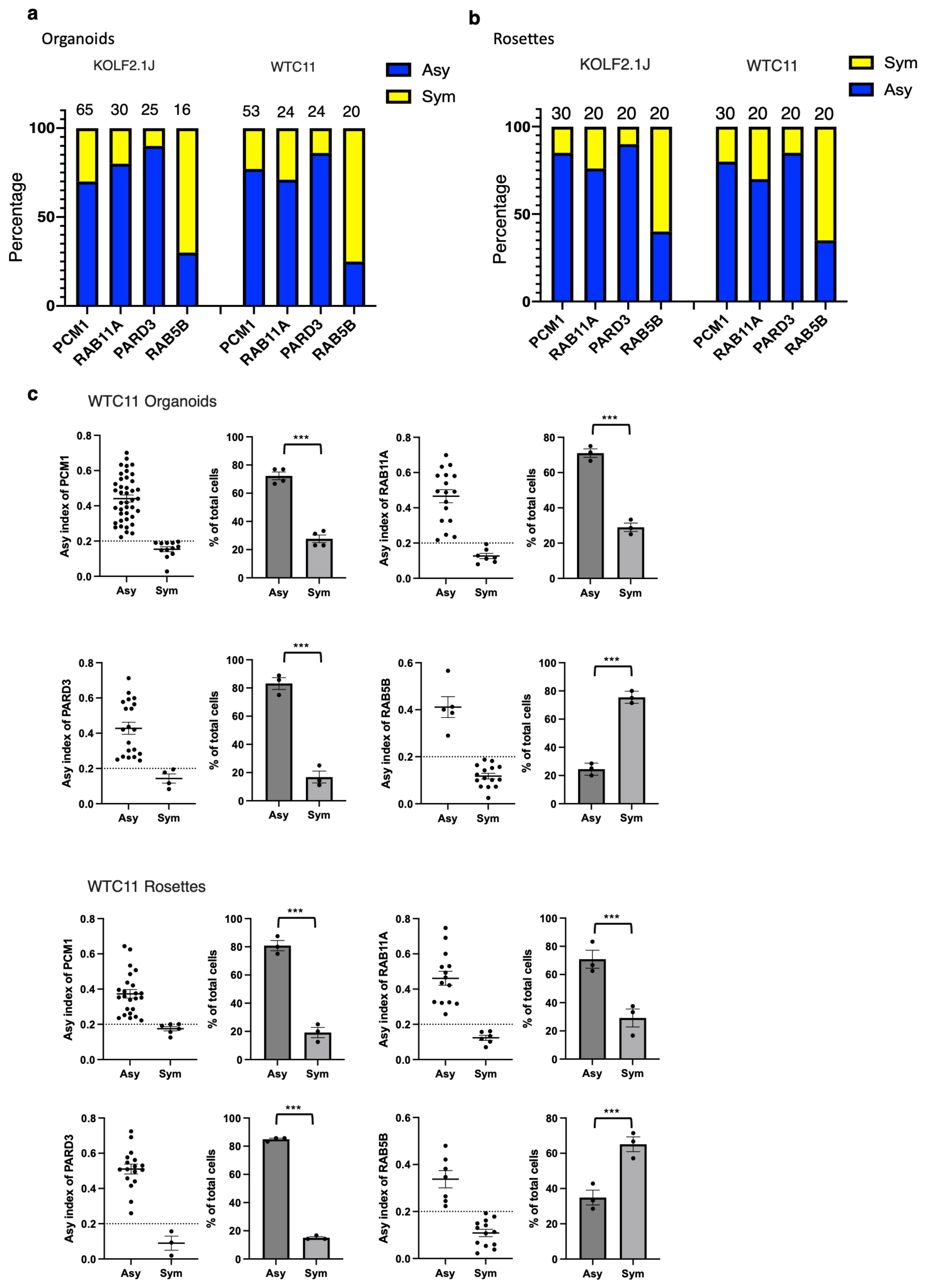
a
Organoids
KOLF2.1J
WTC11
Asy
Sym
65 30 25 16
53 24 24 20
Percentage
0
50
100
PCM1
RAB11A
PARD3
RAB5B
b
Rosettes
KOLF2.1J
WTC11
Sym
Asy
30 20 20 20
30 20 20 20
Percentage
c
WTC11 Organoids
Asy index of PCM1
% of total cells
Asy index of RAB11A
Asy index of PARD3
Asy index of RAB5B
***
Asy
Sym
WTC11 Rosettes

Supplementary Fig 13. **Statistics of asymmetrically vs. symmetrically dividing human neural progenitors. a.** Statistics of PCM1, RAB11A, PARD3, and RAB5B distribution in NPCs at anaphase from both hiPSC line-derived forebrain organoids. **b**. Statistics of PCM1, RAB11A, PARD3, and RAB5B distribution in NPCs at anaphase from both hiPSC lines derived neural rosettes. **c**. Statistics of PCM1, RAB11A, PARD3, and RAB5B asymmetric index in anaphase/telophase NPCs of WTC11 hiPSC lines derived forebrain organoids and neural rosettes. All cells are collected from three independent experiments (n=3). *** $p < 0.001$, two-tailed unpaired t-test. Error bars indicate SD.

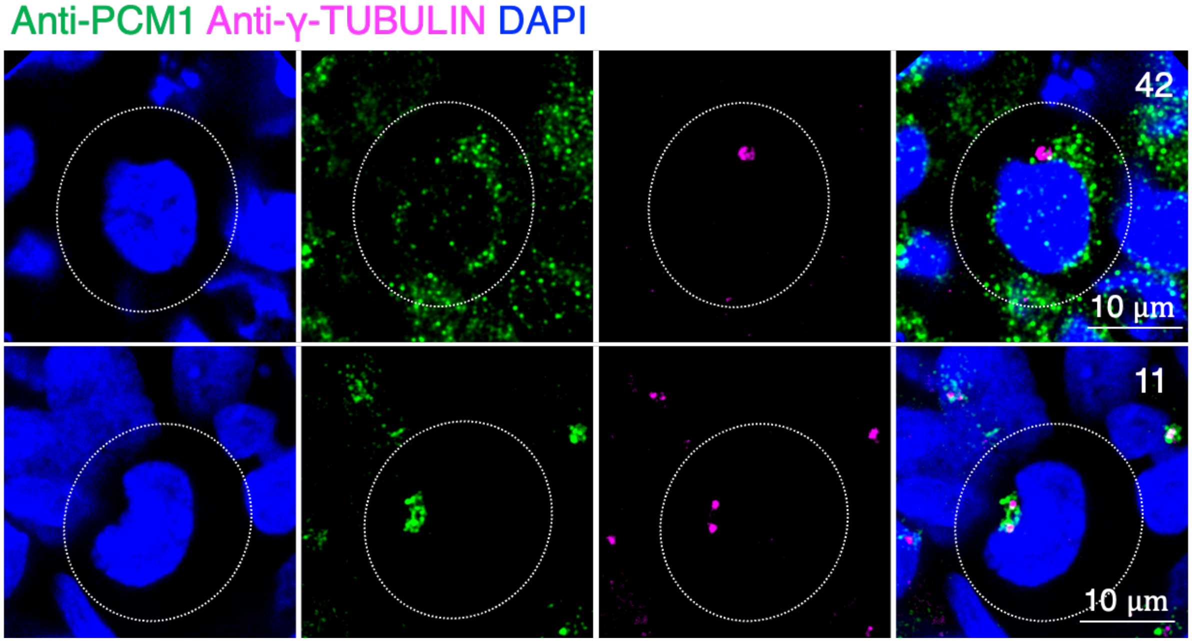

Supplementary Fig 14. Interphase PCM1 distribution patterns in neural progenitor cells (NPCs) of KOLF2.1J hiPSC-derived forebrain organoids. Interphase NPCs with pericentriolar enrichment (upper row, n=42), and tight centrosomal association (lower row, n=11). NPCs were from 8 Day 25 organoids. Scale bars, 10 µm.

Supplemental Video Legends

Suppl. Video 1-3. Time-lapse recordings of RGPs labeled with Pcm1-GFP (green), Myr-Tdt (red), and Dld (Magenta) in the forebrain of ~24 hpf zebrafish embryo, which were shown in Fig. 1h (from top lane to bottom). Video 1 is RGP showed posterior enriched Pcm1-GFP at telophase; Video 2 is RGP showed symmetric Pcm1-GFP at telophase; Video 3 is RGP showed anterior enriched Pcm1-GFP at telophase. Each frame is MIP of 8 z-planes (z step is 1 µm) and scanning interval is 30 sec.

Suppl. Video 4-7. Time-lapse recordings of RGPs labeled with centrin-GFP (green), Myr-Tdt (blue), and Dld (magenta) in the forebrain of ~24 hpf zebrafish embryo, which were shown in Fig. 2a. Video 4 is RGP from control MO embryo; Video 5 is RGP from *pcm1* MO embryo; Video 6 is RGP from *pcm1* KO embryo; Video 7 is RGP from *pcm1* MO +*pcm1* mRNA embryo. Each frame is MIP of 8 z-planes (z step is 1 µm) and the scanning interval is 30 sec. Scale bars indicate 5 µm.

Suppl. Video 8-10. Time-lapse recording of RGPs labeled with H2B-mRFP (red), Par-3-GFP (green in apical side), in *Tg [HuC-GFP]* zebrafish embryonic forebrain, which were shown in Fig. 3d. Video 8 is RGP with P/P division; Video 9 is RGP with P/N division; Video 10 is RGP with N/N division. Each frame is MIP of 10 ~ 15 z-plane (z step is 1 μm) and scanning interval is 6 mins. The time lapse imaging started from 20 hpf and lasted till 36 hpf. Scale bars indicate 10 μm.
