# Supplementary material for: PCM1 coordinates centrosome asymmetry with polarized endosome dynamics to regulate daughter cell fate
Source: Nat Commun. 2025 Nov 28;16:10728. doi: 10.1038/s41467-025-65756-2 (PMC12663461; doi:10.1038/s41467-025-65756-2)
Supplement: Supplementary file 2 — Description of Additional Supplementary Files [file 41467_2025_65756_MOESM2_ESM.pdf]

Title: Supplementary Videos 1-3.

Description: Time-lapse recordings of RGPs labeled with Pcm1-GFP (green), Myr□Tdt (red), and Dld (Magenta) in the forebrain of ~24 hpf zebrafish embryo, which were shown in Fig. 1h (from top lane to bottom).

Video 1 is RGP showed posterior enriched Pcm1-GFP at telophase

Video 2 is RGP showed symmetric Pcm1-GFP at telophase

Video 3 is RGP showed anterior enriched Pcm1-GFP at telophase. Each frame is MIP of 8 z-planes (z step is 1 μm) and scanning interval is 30 sec.

Title: Supplementary Videos 4-7. Time-lapse recordings of RGPs labeled with centrin-GFP (green), Myr□Tdt (blue), and Dld (magenta) in the forebrain of ~24 hpf zebrafish embryo, which were shown in Fig. 2a.

Video 4 is RGP from control MO embryo

Video 5 is RGP from pcm1 MO embryo

Video 6 is RGP from pcm1 KO embryo

Video 7 is RGP from pcm1 MO +pcm1 mRNA embryo. Each frame is MIP of 8 z-planes (z step is 1 μm) and the scanning interval is 30 sec. Scale bars indicate 5 μm.

Title: Supplementary Videos 8-10.

Description: Time-lapse recording of RGPs labeled with H2B-mRFP (red), Par-3- GFP (green in apical side), in Tg [HuC-GFP] zebrafish embryonic forebrain, which were shown in Fig. 3d.

Video 8 is RGP with P/P division

Video 9 is RGP with P/N division

Video 10 is RGP with N/N division. Each frame is MIP of 10 ~ 15 z-plane (z step is 1 μm) and scanning interval is 6 mins. The time lapse imaging started from 20 hpf and lasted till 36 hpf. Scale bars indicate 10 μm.
