# Supplementary material for: PCM1 coordinates centrosome asymmetry with polarized endosome dynamics to regulate daughter cell fate
Source: Nat Commun. 2025 Nov 28;16:10728. doi: 10.1038/s41467-025-65756-2 (PMC12663461; doi:10.1038/s41467-025-65756-2)
Supplement: Supplementary file 13 — Reporting Summary [file 41467_2025_65756_MOESM13_ESM.pdf]

nature portfolio

Corresponding author(s): Xiang Zhao; Loic Royer; Zhiqiang Dong;Su Guo

Last updated by author(s): Sep 16, 2025

Reporting Summary

Nature Portfolio wishes to improve the reproducibility of the work that we publish. This form provides structure for consistency and transparency in reporting. For further information on Nature Portfolio policies, see our Editorial Policies and the Editorial Policy Checklist.

Statistics

For all statistical analyses, confirm that the following items are present in the figure legend, table legend, main text, or Methods section.

n/a	Confirmed	
☐	☒	The exact sample size (*n*) for each experimental group/condition, given as a discrete number and unit of measurement
☐	☒	A statement on whether measurements were taken from distinct samples or whether the same sample was measured repeatedly
☐	☒	The statistical test(s) used AND whether they are one- or two-sided<br>*Only common tests should be described solely by name; describe more complex techniques in the Methods section.*
☐	☒	A description of all covariates tested
☐	☒	A description of any assumptions or corrections, such as tests of normality and adjustment for multiple comparisons
☐	☒	A full description of the statistical parameters including central tendency (e.g. means) or other basic estimates (e.g. regression coefficient) AND variation (e.g. standard deviation) or associated estimates of uncertainty (e.g. confidence intervals)
☐	☒	For null hypothesis testing, the test statistic (e.g. *F*, *t*, *r*) with confidence intervals, effect sizes, degrees of freedom and *P* value noted<br>*Give P values as exact values whenever suitable.*
☒	☐	For Bayesian analysis, information on the choice of priors and Markov chain Monte Carlo settings
☒	☐	For hierarchical and complex designs, identification of the appropriate level for tests and full reporting of outcomes
☒	☐	Estimates of effect sizes (e.g. Cohen's *d*, Pearson's *r*), indicating how they were calculated

Our web collection on statistics for biologists contains articles on many of the points above.

Software and code

Policy information about availability of computer code

Data collection	Micro_Manager 2.0 and Matlab_2023a has been used for data collection
Data analysis	Micro_Manager 2.0, ImageJ, Matlab_2023a, Prism 10, Aydin (DOI: 10.5281/zenodo.5654826. https://github.com/royerlab/aydin), kymopy (https://gitlab.com/bio4212310/kymopy), JaCop of ImageJ.

For manuscripts utilizing custom algorithms or software that are central to the research but not yet described in published literature, software must be made available to editors and reviewers. We strongly encourage code deposition in a community repository (e.g. GitHub). See the Nature Portfolio guidelines for submitting code & software for further information.

Data

Policy information about availability of data

All manuscripts must include a data availability statement. This statement should provide the following information, where applicable:

- Accession codes, unique identifiers, or web links for publicly available datasets
- A description of any restrictions on data availability
- For clinical datasets or third party data, please ensure that the statement adheres to our policy

All data needed to evaluate the conclusions in the paper are present in the paper and/or the Supplementary Materials. The Bulk mRNA-Seq data generated in this study have been deposited in the the NCBI SRA database, (https://dataview.ncbi.nlm.nih.gov/object/PRJNA1159911). Source Data are provided with this paper. The raw gel data and western blot results generated in this study are provided in the Supplementary Information/Source Data file.

Research involving human participants, their data, or biological material

Policy information about studies with human participants or human data. See also policy information about sex, gender (identity/presentation), and sexual orientation and race, ethnicity and racism.

Reporting on sex and gender	NA
Reporting on race, ethnicity, or other socially relevant groupings	NA
Population characteristics	NA
Recruitment	NA
Ethics oversight	NA

Note that full information on the approval of the study protocol must also be provided in the manuscript.

Field-specific reporting

Please select the one below that is the best fit for your research. If you are not sure, read the appropriate sections before making your selection.

- [x] Life sciences
- [ ] Behavioural & social sciences
- [ ] Ecological, evolutionary & environmental sciences

For a reference copy of the document with all sections, see nature.com/documents/nr-reporting-summary-flat.pdf

Life sciences study design

All studies must disclose on these points even when the disclosure is negative.

Sample size	For the zebrafish experiment, we have included the results from more than three embryos for each group in each experiment. For human iPSC cultures, we have used cell cultures from three or more independent experiments. And we have repeated the same experiment with two different iPSC cell lines. We have included the results from three samples or more In each experiment.
Data exclusions	We did not excluded any sample or data from the experiment.
Replication	All experiments in the studies had been repeated with three times or more for applying statistic analysis.
Randomization	Randomization was integral to the experimental design for both model systems. For experiments involving zebrafish (Danio rerio), independent clutches were generated by crossing multiple pairs of adult fish. To ensure a sufficiently large and variable pool for selection, embryos were collected at random exclusively from crosses that yielded more than 200 zygotes. Similarly, for human induced pluripotent stem cell (hiPSC) cultures, cells were randomly assigned to a minimum of six separate wells upon thawing prior to the start of each experiment.
Blinding	To ensure experimental blinding, embryo collections were performed by technicians and samples were numerically coded. A single, standardized protocol was utilized for all experiments conducted on the embryo groups. To preserve the integrity of the blind, distinct experimental tasks, including the culture of brain organoids and neural rosettes, were executed by different researchers.

Reporting for specific materials, systems and methods

We require information from authors about some types of materials, experimental systems and methods used in many studies. Here, indicate whether each material, system or method listed is relevant to your study. If you are not sure if a list item applies to your research, read the appropriate section before selecting a response.

Materials & experimental systems

n/a	Involved in the study	
[ ]	[x]	Antibodies
[ ]	[x]	Eukaryotic cell lines
[x]	[ ]	Palaeontology and archaeology
[ ]	[x]	Animals and other organisms
[x]	[ ]	Clinical data
[x]	[ ]	Dual use research of concern
[x]	[ ]	Plants

Methods

n/a	Involved in the study	
[x]	[ ]	ChIP-seq
[x]	[ ]	Flow cytometry
[x]	[ ]	MRI-based neuroimaging

Antibodies

Antibodies used	Rabbit anti-γ-tubulin [Sigma Cat# T5192, Research Resource Identifier (RRID): AB_261690, 1:500 for immunostaining]. Mouse anti-γ-tubulin (Sigma-Aldrich Cat# T6557, RRID:AB_477584, 1:1000 for immunostaining). Rabbit anti-CEP83 (Sigma-Aldrich Cat# HPA038161, RRID: AB_10674547, 1:500 for immunostaining), Mouse anti-eGFP (Thermo Fisher Scientific Cat# MA1-952, RRID:AB_889471, 1:500 for immunostaining), Rab5b antibody (rabbit polyclonal, Invitrogen Cat# PA5-44574, RRID: AB_2608403, 1:500 for immunostaining), Rab11a (rabbit polyclonal, Thermofisher PO# 715300, RRID: AB_2533987, 1:500 for immunostaining), Chicken anti-NESTIN (NOVUS biotechne, NB100-1604, 1:500 for immunostaining, Rabbit anti-PAX6 (Biolegend, 901901, 1:250 for immunostaining), Mouse anti-PAX6(BD Biosciences, 562249,1:500 for immunostaining), Rabbit Anti-TBR2 (Abcam, ab23345; RRID, AB_778267, 1:200). Anti-HuC/D (mouse monoclonal, Thermofisher PO# A-21271, RRID: AB_221448, 1:500 for immunostaining), Mouse anti-Dld (Abcam, ab73331; RRID, catalog number: AB_1268496; lot GR115501-3, 1:200 dilution for immunostaining); Chicken anti-GFP (Abcam, catalog number: ab13970; RRID:AB_300798, lot GR3190550-20, 1:500 dilution for immunostaining); Rabbit anti-Par-3 (Millipore 07-330; RRID:AB_2101325; lot 3322358, 1:500 for immunostaining); Guinea pig anti-DLIC1-Cter (a gift from Dr. T. Uemura, 1:100 for immunostaining) ; Rabbit anti-Pcm1 (Rabbit antibodies raised against C-terminus of human PCM-1 comprising nucleotides 4993–6095, 1:200 for immunostaining) was provided by Dr. Merdes. Chicken anti-PCM1 antibody was produced in the study and validated by immunostaining and western-blot.
Validation	Rabbit anti-γ-tubulin :https://www.sigmaaldrich.com/US/en/product/sigma/t5192?srsltid=AfmBOoouiZz5EIvU0hVrbfs86tn_xGYqiK9wbI_rPkqbEKc1Ao90FZp8<br>Mouse anti-γ-tubulin: https://www.sigmaaldrich.com/US/en/product/sigma/t6557?srsltid=AfmBOoqtawl-nKzSVoPpHJNY2J5cQjd-3a5bSuT3BNjQV5CU4WR_0WSH<br>Rabbit anti-CEP83: https://www.sigmaaldrich.com/US/en/product/sigma/hpa038161?srsltid=AfmBOopZIKKP6EAk218BmxXIpRBzJAbRm3issEpS-iuj4NZKFxVTMiIY<br>Mouse anti-eGFP: https://www.thermofisher.com/antibody/product/eGFP-Antibody-clone-F56-6A1-2-3-Monoclonal/MA1-952<br>Rab5b antibody: https://www.thermofisher.com/antibody/product/RAB5B-Antibody-Polyclonal/PA5-44574<br>Rab11a antibody: https://www.thermofisher.com/antibody/product/RAB11A-Antibody-Polyclonal/71-5300<br>Chicken anti-NESTIN: https://www.novusbio.com/products/nestin-antibody_nb100-1604?srsltid=AfmBOoqQpFaitUZUOqmbKhSyFEm5s2GHVoaLtVpNzSrQVcy3y-7jLrD5<br>Rabbit anti-PAX6: https://www.biolegend.com/en-gb/products/purified-anti-pax-6-antibody-11511?GroupID=GROUP26<br>Mouse anti-PAX6:https://www.antibodyregistry.org/AB_11152956<br>Rabbit Anti-TBR2 :https://www.abcam.com/en-us/products/primary-antibodies/tbr2-eomes-antibody-ab23345?srsltid=AfmBOor5y_01zjM2aAhE6anHiK_W9Hmouy5jxc5EmNih3lC3ACr5cAVA<br>Mouse anti-HuC/D: https://www.thermofisher.com/antibody/product/HuC-HuD-Antibody-clone-16A11-Monoclonal/A-21271?ef_id=Cj0KCQjwo63HBhCKARIsAHOHV_VPZwsFlinLlt3WEn_93YyBo7ZstkwDA7TdEd9DorD8ItPyd8WautkaAgQAEALw_wcB:G:s&s_kwcid=AL!3652!3!459736943987!!!g!!!10950825775!106531320406&cid=bid_pca_aup_r01_co_cp1359_pjt0000_bid00000_0se_gaw_dy_pur_con&gad_source=1&gad_campaignid=10950825775&gbraid=0AAAAADxi_GQkWrMi9jwPVLHds6ewFG5jk&gclid=Cj0KCQjwo63HBhCKARIsAHOHV_VPZwsFlinLlt3WEn_93YyBo7ZstkwDA7TdEd9DorD8ItPyd8WautkaAgQAEALw_wcB<br>Mouse anti-Dld : https://www.abcam.com/en-us/products/primary-antibodies/deltad-antibody-dld-5c7-2-zdd2-ab73331?srsltid=AfmBOop7nDGO1K87HbEdr7T2fLXw4vLHb1bo5b42LV6dp_08i1-C2gGI<br>Chicken anti-GFP: https://www.abcam.com/en-us/products/primary-antibodies/gfp-antibody-ab13970?srsltid=AfmBOooU80J4BNmuNsqOOLVzFVrU80JrZEH4Q2RMzYkmQ99HFONa_bCj<br>Rabbit anti-Par-3: https://scicrunch.org/resolver/AB_2101325<br>Guinea pig anti-DLIC1-Cter: Satoh, D. et al. Spatial control of branching within dendritic arbors by dynein-dependent transport of Rab5-endosomes. Nat. Cell Biol. 10, 1164-1171 (2008).<br>Rabbit anti-Pcm1: Dammermann, A. & Merdes, A. Assembly of centrosomal proteins and microtubule organization depends on PCM-1. J. Cell Biol. 159, 255-266 (2002).<br>Chicken anti-PCM1: Validated by this study. Supplementary Fig 8a. Western blotting of 1 dpf embryonic lysate (up) and human PCM1 antigen (bottom) with the custom-generated<br>chicken anti-PCM1 antibody

Eukaryotic cell lines

Policy information about cell lines and Sex and Gender in Research

Cell line source(s)	Human induced pluripotent stem cell lines (hiPSC) WTC11 (RRID: CVCL_Y803) and KOLF2.1J (RRID: CVCL_B5P3)
Authentication	All experiments using human stem cell are approved by UCSF Gamete, Embryo and Stem Cell Research Committee (GESCR).
Mycoplasma contamination	All cells lines are negative for Mycoplasma tests.
Commonly misidentified lines (See ICLAC register)	NA

Animals and other research organisms

Policy information about studies involving animals; ARRIVE guidelines recommended for reporting animal research, and Sex and Gender in Research

Laboratory animals	Zebrafish AB strain

Wild animals	The study did not involved any wild animals.
Reporting on sex	We used zebrafish embryos younger than 2 day post fertilization. At that time, zebrafish embryos have not yet sexually differentiated.
Field-collected samples	The study did not included any field-collected samples.
Ethics oversight	All animal experiments were approved by the Institutional Animal Care and Use Committee (IACUC protocol no. AN206836) at the University of California, San Francisco, USA.

Note that full information on the approval of the study protocol must also be provided in the manuscript.

Plants

Seed stocks	Not applicable
Novel plant genotypes	Not applicable
Authentication	Not applicable
